# Rare chondrosarcoma of the breast treated with quadrantectomy instead of mastectomy: A case report

**DOI:** 10.3892/ol.2014.2803

**Published:** 2014-12-17

**Authors:** VITTORIO PASTA, DANIELA SOTTILE, PAOLO URCIUOLI, LUCA DEL VECCHIO, FILIPPO CUSTURERI, VALERIO D’ORAZI

**Affiliations:** Department of Surgical Sciences, Sapienza University, Rome I-00161, Italy

**Keywords:** chondrosarcoma, sarcoma, breast, surgery, mastectomy

## Abstract

Breast chondrosarcoma is a rare sarcoma that mainly occurs in females >50 years old. To the best of our knowledge, only 16 cases were reported in the literature prior to 2013 and all patients were surgically treated by mastectomy, with or without lymphadenectomy, which was occasionally preceded by neoadjuvant chemotherapy. However, the literature does not report the benefit of mastectomy compared with a more conservative surgery. The present study reports a novel case of extraskeletal chondrosarcoma of the breast. A 63-year-old female patient presented with a neoplasm localized in the upper-outer quadrant of the right breast. The palpable lesion with sharp margins was a firm parenchymatous mass, which was confirmed by ultrasonography and mammography. The patient underwent conservative quadrantectomy instead of mastectomy, followed by post-surgical chemotherapy. A positron emission tomography scan performed five months subsequent to the surgery revealed no remnants of the disease. The patient underwent a strict clinical and instrumental follow-up, and two and half years after surgery, there are no signs of recurrent disease. In conclusion, the present case is currently one of the two cases in which a more conservative quadrantectomy was performed, instead of mastectomy. This surgical approach did not lead to metastasis and resulted in a good follow-up for the patient.

## Introduction

Sarcomas are tumors that rarely occur in the breast, accounting for 0.5–1% of all breast cancers (1). Sarcomas arise from the stromal tissue of the mammary gland and if angiosarcoma, which has a more severe prognosis, is excluded, the course of the disease is not significantly different compared with tumors arising from other sites (2). Chondrosarcoma is a rare type of sarcoma that, to the best of our knowledge, was reported in only five cases in the literature prior to 2001 (3–6) and 11 cases in the literature between 2002 and 2013 (7–17), and mainly occurs in females >50 years old. Macroscopically, chondrosarcoma appears as a whitish-gray, round mass with regular margins and necrotic, hemorrhagic or cystic areas that is characterized by rapid growth, although the sarcoma rarely invades locally or metastasizes to lymph nodes (9–13,18,19). Chondrosarcoma is microscopically characterized by chondroid lacunae, in which numerous chondroblasts exhibit hyperchromatic nuclei and the absence of epithelial areas or other stromal components, with a high mitotic division rate. There is also an absence of estrogen, androgen or HER2 receptors, with a strong expression of the S100 protein, cytokeratin and the membrane epithelial antigen (1). Ultrasonography reveals a hyperechogenic formation, with a polylobated shape, while mammography exhibits a round hyperdense mass with regular margins, which may simulate a benign lesion (20). The differential diagnosis of chondrosarcoma is usually between malignant phyllodes tumor (PT), metaplastic carcinoma (MC), in which a significant percentage of metaplastic elements (>10%) are present, and matrix-producing metaplastic carcinoma (MP-MC), a rare cancer of the breast characterized by osteoid and chondral matrices and the presence of carcinomatous features (20,21). The mainstay of treatment for sarcoma of the breast, as with sarcomas at other sites, is a surgical procedure combined with mastectomy (20). The present study describes a novel case of extraskeletal chondrosarcoma of the breast that was treated with a more conservative surgical treatment instead of mastectomy, as an innovative surgical approach. Written informed consent was obtained from the patient.

## Case report

**Figure 1:**
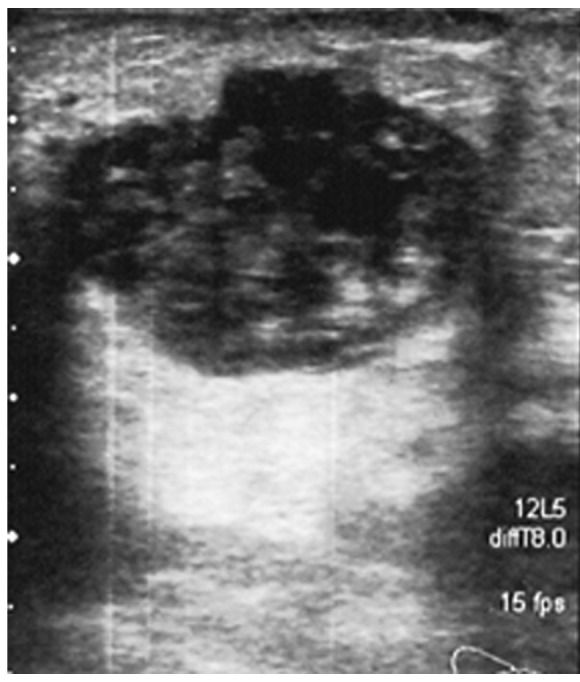
Ultrasonography of the tumor mass. The image reveals the presence of a hypoechoic solid neoformation with jagged edges and a maximum diameter of ~3 cm.

**Figure 2:**
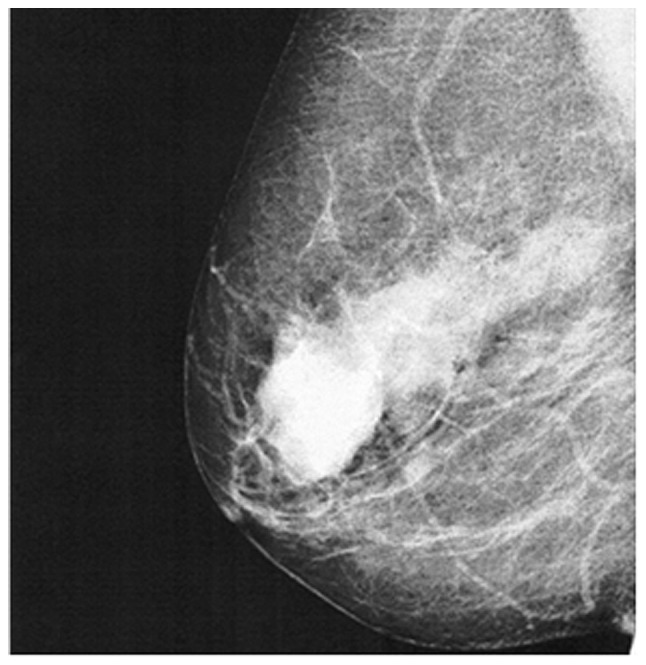
Mammography of the right breast. The images reveal upper-outer para-areolar oval opacity with partially shaded contours, and an adequate margin of healthy perilesional tissue.

A 63-year-old female presented to Umberto I Hospital, Sapienza University (Rome, Italy) with a neoplasm localized in the upper-outer quadrant of the right breast (RUO). The palpable lesion was a firm parenchymatous mass that was mobile on the superficial and deep planes, exhibited sharp margins and a poor homogeneous surface and was not painful. There was no evident axillary lymphadenopathy. An X-ray mammogram revealed the enhancement of a previously observed opacity in the superolateral quadrant, with partial sharp margins. Using core biopsy, the mass was determined to be a mammary parenchyma in continuity with hypercellular condroid tissue, with the presence of atypical, occasionally polynuclear elements, and rare mitosis. Ultrasonography revealed the presence of a hypoechoic, solid neoformation with jagged edges and a maximum diameter of ~3 cm (Fig. 1). Mammography revealed an upper-outer para-areolar oval opacity with partially shaded contours that demonstrated the presence of an adequate margin of healthy perilesional tissue (Fig. 2). The formation was delimited and split by fibrous stroma, in which ductal structures and ectatic vessels were present. The tumor appeared to be a malignant mesenchymal neoplasm with chondroid differentiation, although it could not be excluded that the formation was a component of a biphasic lesion similar to phyllodes tumors. Considering the size of the lesion, the overall dimensions of the breast and the lack of literature reporting a genuine benefit of mastectomy compared with more conservative surgery (Table I), it was decided to refer the patient for a wide RUO quadrantectomy, to include any skip metastasis. For an improved examination, tissue samples of the resection margins were separately analyzed by a pathologist.

### Histopathological results

The gross post-surgical examination revealed a mammary-parenchymal section, 6.5×4.5×5 cm in size, covered by 4.1 cm of skin. Following the cut, a round-shaped neoplasm was observed, possessing a maximum diameter of 3 cm and exhibiting gelatinous and tense-elastic regions. The biopsy specimen from the neoplasm revealed the proliferation of mesenchymal cells with round nuclei, eosinophilic cytoplasm and ill-defined boundaries. The neoplastic elements were arranged in groups of varying sizes, in the context of a layer with a basophilic myxoid appearance. A chondroid-type layer was present at the periphery of the lesion, with a star-shaped appearance and pleomorphic nuclei. Certain figures were suggestive of invasion, however, no perilesional venous vessel invasion was detected. The mitotic index was seven out of 10 fields of high magnification. The tumor possessed large areas of necrosis. The neoplastic elements exhibited 50% of the proliferation index, as evaluated by Ki-67 staining. The remaining parenchyma demonstrated a nodular lesion with a diameter of 1.2 cm attributable to stromal fibrosis, in the absence of further significant atypia. All resection margins were separately analyzed by a pathologist and were found to be free from cancer-associated elements. A diagnosis of chondrosarcoma of the breast was finally made.

### Post-surgical treatments

**Figure 3:**
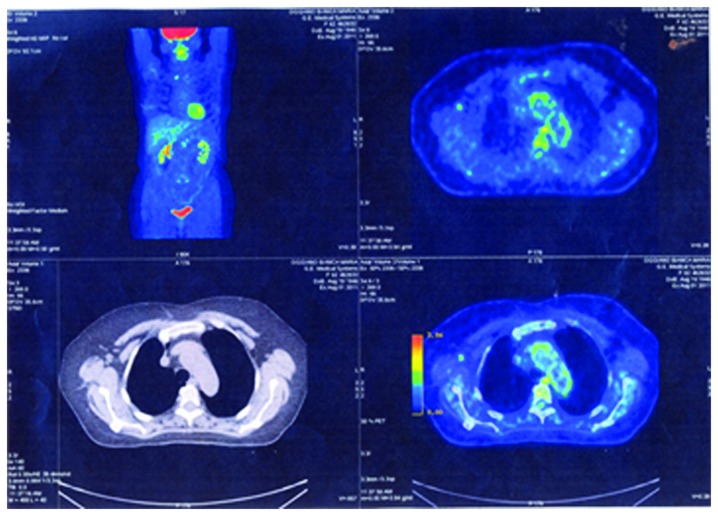
Positron emission tomography examination performed approximately five months after surgery, through which the presence of recurrence was excluded.

Subsequent to surgery there was a discussion between the various consulted oncologists on the necessity of further treatments, as the role of chemotherapy and radiotherapy in primary breast chondrosarcoma is unresolved (1). Despite the possibility that adjuvant therapy may decrease the rates of local and systematic recurrence, the literature is lacking in significant information regarding the benefit of post-surgery chemo- or radiotherapy in the face of the side-effects of those therapies, due to the rarity of this disease and the small number of cases reported (1). It was decided, with the consent of the patient, to administer five cycles of standard radiotherapy on the residual breast tissue (50 Gy) and operative site (10 Gy), used only as a complement to radical surgery, and six cycles of precautionary chemotherapy for six weeks, with epirubicin (120 mg per cycle) and ifosfamide (2,950 mg per cycle), as previously reported (1). A positron emission tomography scan performed five months after the surgery excluded the presence of remnants of the disease (Fig. 3). Therefore, whilst the patient continues to undergo a strict clinical and instrumental follow-up two and half years after surgery, there are no signs of recurrent disease.

## Discussion

The present study describes a novel case of extraskeletal chondrosarcoma of the breast that was treated with a more conservative quadrantectomy instead of mastectomy, as an innovative surgical approach. The benefit of performing quadrantectomy instead of mastectomy is not addressed in the literature (1,2). Between 1967 and 2001, only five cases of chondrosarcoma of the breast were reported in the literature, and all were treated with mastectomy (3–6). The present review revealed that between 2001 and 2013 only 11 other cases were described, as summarized in Table I. All the 16 studies, consisting of 15 female patients and one male patient, reported mastectomy as the surgical treatment choice. In four cases mastectomy was associated with lymphadenectomy and in one case mastectomy was preceded by neoadjuvant chemotherapy (Table I). The choice of mastectomy was dictated not only by a strictly local situation of the lesion, but also by the lack of previous studies, due to the rarity of the lesion, and by an old interpretation of the surgical approach to sarcoma at this particular site.

Although surgery remains the gold standard for the treatment of breast sarcoma, and chondrosarcoma in particular (1), there is no uniformity of view on the most effective type of surgery that may justify the requirement for radical intervention, such as mastectomy, compared to surgery with wide tumor-free margins (20). According to a study performed by Zelek *et al* (22), and another previous study (23), mastectomy for sarcomatous malignancy should not be associated with axillary lymphadenectomy, as the sarcoma does not exhibit a tendency to spread through lymphatic system, but mainly through the haematogenous route. Furthermore, the lymphectomy exposes the patient to greater morbidity without a real benefit in terms of disease-free and overall survival (20). Thus, lymphectomy should be indicated only if the lesion is associated with an epithelial component or when all breast quadrants are involved, in particular the upper-outer (superolateral) quadrants (20).

In previous years, the surgical approach to all sarcomas, and in particular to those of the breast, has been reevaluated as it has been found that an extensive surgical procedure may be considered adequate, providing that the curative wide margins of healthy peritumoral tissue are sufficiently respected, to ensure that the margins include any skip metastasis with the excision (2,24,25). At present, only two factors have been demonstrated to affect the outcome of surgery, the extent of the tumor and the margins of excision. Tumors >5 cm in size result in a poorer prognosis compared with those of smaller dimensions (20). In addition, the majority of authors agree that a resection margin of 1 cm is sufficient for small and localized sarcomas, and this approach is compatible with a conservative surgery. Therefore, in the present study, it was decided to treat the chondrosarcoma of the breast with surgery, in consideration of the size of the lesion, and, in particular, to use a more conservative quadrantectomy instead of mastectomy, as a novel surgical approach. This approach was chosen with the consideration that a greater efficacy has not been proven or demonstrated in patients treated with mastectomy in terms of overall and disease-free survival compared with patients treated by adequate quadrantectomy.

The present treatment strategy was in agreement with a previous retrospective study revealing that, for sarcomas of the breast, the radical treatment of mastectomy did not offer significant survival benefits compared with the wide excision option of quadrantectomy (20). By contrast, the study revealed a more severe prognosis for the patients that underwent simple lumpectomy. Notably, at the time of writing, a novel case report was published in the literature that used a similar conservative breast surgery approach (26), supporting the present approach of performing quadrantectomy instead of mastectomy.

## Figures and Tables

**Table I:** Review of the cases of chondrosarcoma of the breast reported in the literature between 2001 and 2013.

First author, year (reference)	Gender	Age, years	Tumor site, breast	Method used for diagnosis	Therapy	Follow-up
Errarhay *et al*, 2013 (7)	Female	24	Right	Mammography and tumorectomy	Mastectomy	NR
Mujtaba *et al*, 2013 (13)	Female	40	Right	Mammography and CT	Mastectomy	
Badyal *et al*, 2012 (8)	Male	80	Right	FNAC	Mastectomy and axillary lymphadenectomy	Yes
Patterson *et al*, 2011 (12)	Female	52	Left	FNAC and core biopsy	Mastectomy and RT	Yes
Lakshmikant *et al*, 2010 (14)	Female	42	Left	Core biopsy	Mastectomy	NR
Bhosale *et al*, 2010 (15)	Female	45	Right	Tumorectomy and axillary lymph node FNAC	Mastectomy and axillary lymphadenectomy, RT-CHT	Yes
De Padua *et al*, 2009 (11)	Female	56	Right	FNAC	Mastectomy and RT	NR
Gurleyik *et al*, 2009 (16)	Female	52	Right	Ultrasonography, mammography, FNAC and tumorectomy	Mastectomy and axillary lymphadenectomy	NR
Gupta *et al*, 2006 (10)	Female	46	Left	FNAC	Mastectomy	NR
Verfaille *et al*, 2005 (17)	Female	77	Right	Ultrasonography, mammography and Tru-cut needle biopsy	Mastectomy	NR
Gupta *et al*, 2003 (9)	Female	46	Left	FNAC	Mastectomy, neoadjuvant CHT, RT and lymphadenectomy	NR

CT, computed tomography; FNAC, fine-needle aspiration cytology; RT, radiotherapy; CHT, chemotherapy; NR, not reported.
